# Inflammasome Activation Is Critical to the Protective Immune Response during Chemically Induced Squamous Cell Carcinoma

**DOI:** 10.1371/journal.pone.0107170

**Published:** 2014-09-30

**Authors:** Thais Helena Gasparoto, Carine Ervolino de Oliveira, Luisa Thomazini de Freitas, Claudia Ramos Pinheiro, Juliana Issa Hori, Gustavo Pompermaier Garlet, Karen Angélica Cavassani, Roxana Schillaci, João Santana da Silva, Dario Simões Zamboni, Ana Paula Campanelli

**Affiliations:** 1 Department of Biological Sciences - Microbiology and Immunology, Bauru School of Dentistry, University of São Paulo, Bauru, SP, Brazil; 2 Department of Stomatology - Oral Pathology, Bauru School of Dentistry, University of São Paulo, Bauru, SP, Brazil; 3 Departament of Pathology, Medical School, University of Michigan, Ann Arbor, Michigan, United States of America; 4 Laboratorio de Mecanismos Moleculares de Carcinogénesis, Instituto de Biología y Medicina Experimental, Consejo Nacional de Investigaciones Científicas y Técnicas de Argentina, Buenos Aires, Argentina; 5 Department of Biochemistry and Immunology, Ribeirão Preto Medical School, University of São Paulo, Ribeirão Preto, SP, Brazil; 6 Department of Cell Biology, Ribeirão Preto Medical School, University of São Paulo, Ribeirão Preto, SP, Brazil; University of California Merced, United States of America

## Abstract

Chronic inflammation affects most stages of tumorigenesis, including initiation, promotion, malignant differentiation, invasion and metastasis. Inflammasomes have been described as involved with persistent inflammation and are known to exert both pro and antitumour effects. We evaluated the influence of apoptosis-associated speck-like protein containing a caspase recruitment domain (ASC) and caspase (CASP)-1 in the antitumor immune response using a multistage model of squamous cell carcinoma (SCC) development. Absence of ASC and CASP-1 resulted in an earlier incidence and increased number of papilloma. Loss of inflammassome function in mice resulted in decreased presence of natural killer (NK), dendritic (DC), CD4^+^, CD8^+^ and CD45RB^+^ T cells in the tumor lesions as well as in lymph nodes (LN) compared with WT mice. Increased percentage of CD4^+^CD25^+^Foxp3^+^ T cells was associated with association with inflammasome loss of function. Moreover, significant differences were also found with neutrophils and macrophage infiltrating the lesions. Myeloperoxidase (MPO), but not elastase (ELA), activity oscillated among the groups during the SCC development. Levels of proinflammatory cytokines IL-1β, IL-18, Tumor Necrosis Factor (TNF)-α and Interferon (IFN)-γ were decreased in the tumor microenvironment in the absence of inflammasome proteins. These observations suggest a link between inflammasome function and SCC tumorigenesis, indicating an important role for inflammasome activation in the control of SCC development.

## Introduction

An effective immune response is important to restrict the development of the disease, mainly via induction, function, and regulation of effector lymphocytes [1], [2]. Cancer immunosurveillance is described as lymphocytes acting as sentinels in recognizing and eliminating continuously arising transformed cells, and it is exerted mostly by cytotoxic CD8^+^ T-lymphocytes (CTL) while specific immune suppression is associated with tumor malignancy and progression [2]–[4]. CD4^+^ T cells either contribute to tumor destruction or facilitate tumorigenesis depending on their phenotype and function [5]. Among the different subpopulations, Type 1 CD4^+^ T cells (Th1) facilitate tumor rejection by assisting in the CTL response. On the other hand, several studies have shown that CD4^+^ T regulatory cells (Tregs) promote tumor progression by inhibiting the functions of CD8^+^ T cells and natural killer (NK) cells and that the accumulation of Treg cells is associated with a poor prognosis [5]–[7]. In spite of crucial role of T cells in cancer immunosurveillance, the innate immunity also plays an important part in the development and control of tumor response [8], [9]. Neutrophil Elastase (ELA) and Myeloperoxidase (MPO) have also been directly or indirectly related to the cancer immunoediting and metastasis [8], [9]. In recent years, a molecular platform assembled in the host cytosol, named inflammasome, has been described. The proteolytic maturation of inflammatory cytokines IL-18 and IL-1β may result from inflammasome activation [10]. Inflammasomes are a set of intracellular protein complexes that enable autocatalytic activation of inflammatory caspases, as caspase-1, which drive host immune responses by releasing pro-inflammatory cytokines and inducing pyroptosis [11]. Their activation occurs in response to pathogens (PAMPs) and stress or danger signals (DAMPs) promoting the maturation of cytokines of the IL-1 family: IL-1β and IL-18 [12], [13]. Different studies have been showing the important role of IL-18 as the primary IFN-γ inducing cytokine and responsible to promote a Th1 response [14], [15].

Besides promotion of a Th1 response, IL-18 has been described, as along with IL-12 and other cytokines, as being important for NK cells action upon aberrant cells [16], [17]. Thus, we rationalize if the tumor cells do not elicit the inflammasome mediated innate immune response they would be regarded as self and therefore will escape of the T cell immune surveillance and immunogenic cell death (ICD) [18], [19]. ICD markers are released from apoptotic and necrotic cells (e.g. cancer cells) to stimulate the innate and adaptive immune responses. Cell death products such as damage-associated molecular pattern molecules (DAMPs) play an essential role in addressing that process [18], [19]. Interaction of these DAMPs with various receptors on the surface of immune cells, as well as stromal cells, is required to evoke the ICD-induced anti-tumor immune responses [18].

IL-1β is a potent pro-inflammatory cytokine, whose levels are increased in the cancer conditions [10], [12]. The transcription of pro-IL-1β is induced by the activation of the transcription factor nuclear factor-κB (NF-κB), whereas pro-IL-18 is constitutively expressed and its expression is increased after cellular activation [11], [13]. Therefore, both pro-inflammatory cytokines are controlled by two checkpoints: the transcription, and maturation and release [13]. In turn, the activation of IL-1β by CASP-1 leads to the recruitment and the activation of immune cells at the site of tissue damage [13]. Several NOD-like receptors (NLRs) act as sensor molecules that can trigger the formation of inflammasomes. Most of the inflammasomes that have been described to date contain NLRs: NLRP1 (NOD-, LRR- and pyrin domain-containing 1), NLRP3, NLRP6, NLRP7, NLRP12 and NLRC4 (13, 20-23). AIM2 and the NLRP proteins, with the exception of NLRP1, require an adaptor protein - Apoptosis-associated speck-like protein containing a caspase recruitment domain (ASC)- to activate CASP-1 [13], [20]–[25]. The influence of inflammasome in the carcinoma development has been shown to have contrasting roles in the interplay between malignant cells and the tumor microenvironment [5], [10], [24]–[26]. Studies have shown different role of ASC according to the type of tumor [26], [27]. Because the inhibition of inflammasomes has been shown to have profound effects on carcinogenesis and tumor progression [10], we decided to investigate the inflammasome function in the development of experimentally induced murine squamous cell carcinoma (SCC).

## Materials and Methods

### Mice

Experimental animals were cared for in accordance with of the National Council of Health and the University of São Paulo (Bauru, São Paulo, Brazil) committee guidelines. C57BL/6 mice [Wild-type (WT)], ASC (ASC-KO), and CASP-1-deficient (CASP-1-KO) mice (6–8 weeks old) were obtained from Ribeirão Preto Medical School, University of São Paulo. Each mouse was housed in an isolated cage, and food and water were provided ad libitum. All experimental procedures involving animals in this study were reviewed and approved by the Animal Research Ethics Committee of the Bauru School of Dentistry, University of São Paulo.

### DMBA/PMA-induced skin carcinogenesis initiation-promotion experiments

Eight-week-old female mice were topically treated with four doses of 7,12-dimethylbenz[a]-anthracene (DMBA) (25 µg in 200 µl of acetone) and biweekly doses of phorbol 12-myristate 13-acetate (PMA) (200 µl of a 10^−4^ M solution in acetone) for 20 weeks. Both DMBA and PMA (Sigma–Aldrich) solutions were applied using a pippete (200 µl) in acetone onto the shaved back skin Papilloma incidence was monitored every two days. Papillomas were characterized by folded epidermal hyperplasia protruding from the skin surface, and carcinomas were characterized as endophytic tumors presenting as plaques with an ulcerated surface. Samples were collected at different time-points after initiation and were processed as described below.

### Histological analysis

Tissue samples were collected from tumour sites and fixed with 10% (vol/vol) formalin for 6 hours at room temperature. The tissues were subsequently dehydrated in ethyl alcohol followed by washes in xylol and were then embedded in paraffin. Each sample was sectioned into 5- to 7-µm-thick slices that were dried onto slides and stained with hematoxylin and eosin.

### Isolation of leukocytes

To characterize the leukocytes in the tumor site, biopsies of skin lesions from mice were collected and incubated for 1 h at 37°C in RPMI 1640 medium containing 50 µg/ml of a collagenase CI enzyme blend (Boehringer Ingelheim Chemicals). The tissues were subsequently dissociated for 4 min in RPMI 1640 with 10% serum and 0.05% DNase (Sigma-Aldrich) using a Medmachine (BD Biosciences) according to the manufacturer's instructions. The tissue homogenates were filtered using a 30-µm cell strainer (Falcon; BD Biosciences). Leukocyte viability was evaluated by Trypan blue exclusion, and these cells were subsequently used for cell activation and immunolabeling assays. Isolated cells were counted using Newbauer chamber. The total number of cells was normalized against the events acquired in flow cytometry.

### Antibodies and flow cytometry analysis

For immunostaining, APC-, PerCP-, PE- and FITC conjugated Abs against CD3, CD4, CD8, CD11c, CD19, CD25, CD28, CD45RA, CD45RB, CD62L, NK1.1, F4/80, GR-1 and the respective isotype controls were used (BD Biosciences). PE-CD209/DC SIGN was acquired from EBiosciences. The intracellular detection of Foxp3 in leukocytes was performed using Cytofix/Cytoperm and Perm/Wash buffer from BD Biosciences according to the manufacturer's instructions. Briefly, the cells were labeled with Abs to the cell surface, such as FITC anti-CD25 and PerCP-conjugated anti-CD4. Following the staining of surface markers, the cells were fixed, permeabilized and stained with PE-labeled anti-mouse Foxp3 (MACS Miltenyl Biotech), The samples were acquired on a FACSort flow cytometer, and the data were analyzed using CellQuest software (BD Biosciences).

### MPO and ELA activity quantification

MPO and ELA activities were measured in the supernatants from lesions. Lesion sample supernatants were obtained by disaggregating the tissue through treatment with RPMI 1640 medium containing 0.25% collagenase (Worthington) and were frozen at -80°C until analysis. The total protein concentration was measured using a Quick Start Bradford Protein assay kit (Bio-Rad, CA, USA). MPO activity in the supernatants was assayed by measuring the change in absorbance at 450 nm using 3,3′,5,5′ tetramethylbenzidine (50%) and H_2_O_2_ (50%) (Opteia Set B; BD Biosciences, San Diego, CA, USA). The reaction was stopped after 15 min using phosphoric acid (1M).

For the ELA analysis, supernatants were individually incubated with insoluble elastin-Congo Red dye (E0502-5G; Sigma- Aldrich) diluted in 100 mM Tris–HCl buffer (pH 8.8) containing 0.01% sodium azide (Sigma- Aldrich) in a 96-well plate. The optical absorption of the hydrolysis product was read with a Microplate Reader (Bio-Rad Model 680; Bio-Rad Laboratories, Hercules, CA, USA) at 450 nm after 24 h incubation at 37°C. ELA activity was calculated from a standard curve of elastase from human leucocytes (Sigma-Aldrich) and expressed in units/ml.

### Cytokine assays

IL-1β, IL-10, IFN-γ and TNF-α levels in the supernatants of samples were individually quantified using ELISA kits, according to the manufacturer's instructions (BD Pharmingen Corp., San Diego, CA). IL-18 was quantified through Mouse IL-18 Platinum ELISA (Ebiosciences) kit according to the manufacturer's instructions.

### Statistical analysis

The results are expressed as the mean ± SD, and statistical analysis was performed using two-way ANOVA followed by Bonferroni's test (GraphPad software 4). p≤0.05 was considered to indicate statistical significance.

## Results

### Inflammasome loss of function accelerated the appearance of the SCC in mice

**Figure 1:**
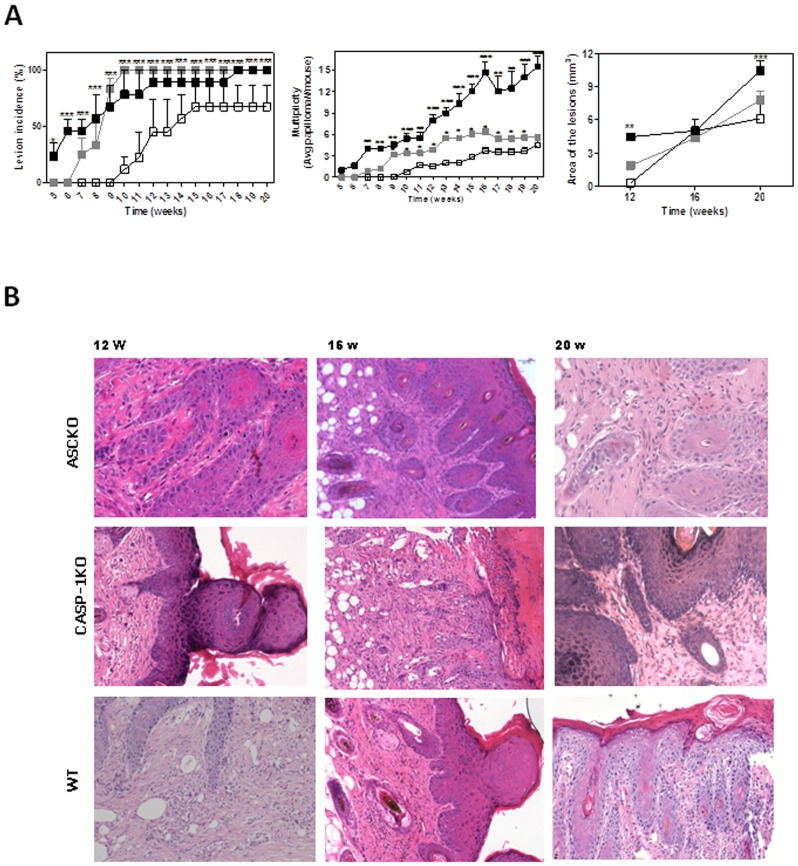
Absence of inflammasome molecules induced an increase of papilloma incidence and volume during SCC development. ASC-KO (gray square), CASP-1-KO (black square) and WT (white square) mice were treated according to a chemical carcinogenic protocol using DMBA and PMA for 20 weeks. (A) The incidence, average number and volume of papillomas were determined. Data shown represent mean ± SEM; *p≤0.05, ** *p*≤0.01, *** *p*≤0.001 compared with WT mice. Representative data are show from at least three independent experiments. (B) Representative haematoxylin and eosin staining of skin section from 12, 16 and 20 weeks after SCC induction. Scale bars represent 50 uM.

To investigate the effects of inflammasome signaling on tumor initiation and development, we used a modified well-established protocol to induced skin tumorigenesis on WT, ASC-KO and CASP-1-KO mice [26]. Inflammasome loss of function significantly enhanced papilloma incidence, rate and size, when compared with WT mice (Fig. 1A). Macroscopically visible papillomas were detected at the 5^th^ week of TPA application on CASP-1-KO, at the 7^th^ week on ASC-KO, and only at the 10^th^ week on WT mice (Fig. 1A). In WT group, all mice developed papilloma by the 20^th^ week of DMBA/TPA application (Fig. 1A). In contrast, loss of inflammasome function enhanced the process of papilloma development. All ASC-KO mice developed visible papilloma by the 10^th^ week after carcinogenic induction (Fig. 1A) while all CASP-1-KO mice developed papilloma by the 18^th^ week (Fig. 1A). The average tumor volume per mouse in the CASP-1-KO mice was significantly higher compared with the WT group (Fig. 1A). No significant differences were found relating tumor volume between ASC-KO and WT mice (Fig. 1A). Moreover, CASP-1-KO group exhibited significantly more lesions than the WT group from 7^th^ week until 20^th^ week while ASC-KO showed lesions from the 9^th^ week until the 19^th^ week (Fig. 1A). The proportion of mice presenting papilloma and/or tumor is shown in the Table 1.

**Table 1:** Histologic analysis of tumors from wild type and inflammasome-deficient mice.

Tumor	WT (%)	ASC-KO (%)	CASP-1-KO (%)
Papilloma	8/11 (73.3)	11/11 (100)	11/11 (100)
SCC	1/11 (9.1)	7/11 (63.6)	10/11 (90.9)

Wild type (WT), ASC and CASP-1 deficient mice were followed until 20 weeks after skin carcinogenesis, and results from histological analysis were tabulated and compared.

By the 12^th^ week, histological analysis of lesion samples from theinflammasome-deficient groups demonstrated typical papilloma characteristics (Fig.1B). There was discrete inflammation around the skin appendages which were less numerous than in the control tissue. Each finger like projection was lined by stratified squamous epithelium and contained a thin central connective tissue. The histological analysis also demonstrated that the groups with inflammasome loss of function presented moderated inflammatory infiltrate and some mitotic cells (Fig. 1B) that remained at the 16^th^ week (Fig. 1B). We identified epithelial hyperplasia, localized regions with hyperkeratosis, presence of atypical mitotic cells exhibiting dense chromatin and epithelial islets (Fig. 1B). At the 20^th^ week, hyperkeratosis, epithelial hyperplasia, epithelial crest, islets and invasive cords of malignant squamous epithelial cells arose independently from epithelium surface inside the connective tissue in KO mice (Fig. 1B). There was also an increased inflammatory infiltrate, atypical mitosis areas, poor differentiated pattern and presence of pseudoglandular appendages (Fig. 1B). The histological analysis of skin samples from WT mice, at the 12^th^ week, showed desmosomes on outer surface of the epithelium, presence of inflammatory cells in the stratum spinosum and normal keratinocytes in stratum corneum (Fig. 1B). At the 16^th^ week, less keratin alteration, moderate inflammatory infiltrate, presence of mitotic cells, and a lack of epithelial islet formation was also observed (Fig. 1B). At the 20^th^ week, a pronounced presence of inflammatory cells, epithelial islets, mitosis and moderate dysplasia were observed in WT samples (Fig. 1B). Altogether, these results demonstrate that inflammasome activation during tumorigenesis can attenuate tumor progression.

### Inflammasome activation regulates the inflammatory infiltrate of SCC redirecting it to a tumor suppressor phenotype

**Figure 2:**
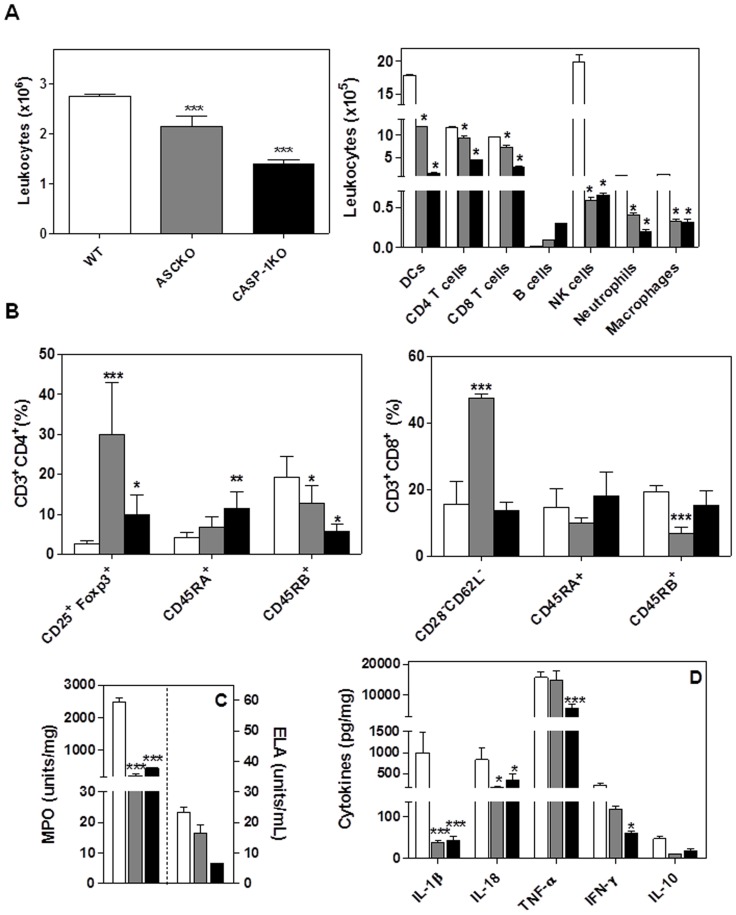
Characterization of SCC lesions in the absence of inflammasome after 12 weeks. (A) The absolute number and phenotype of leukocytes in the tumor lesions were determined by flow cytometry. (B) Flow cytometry analyses of CD25^+^Foxp3^+^, CD45RA^+^, CD45RB^+^ and CD28^−^CD62L^−^ expression on CD4 e CD8 T cells. (C) Myeloperoxidase (MPO) and elastase (ELA) activity in tumor microenvironment. (D) Cytokines profile in tumor microenvironment at 12 weeks after SCC induction. Values are mean ± SEM; *p≤0.05, *** *p*≤0.001 compared with WT mice.

**Figure 3:**
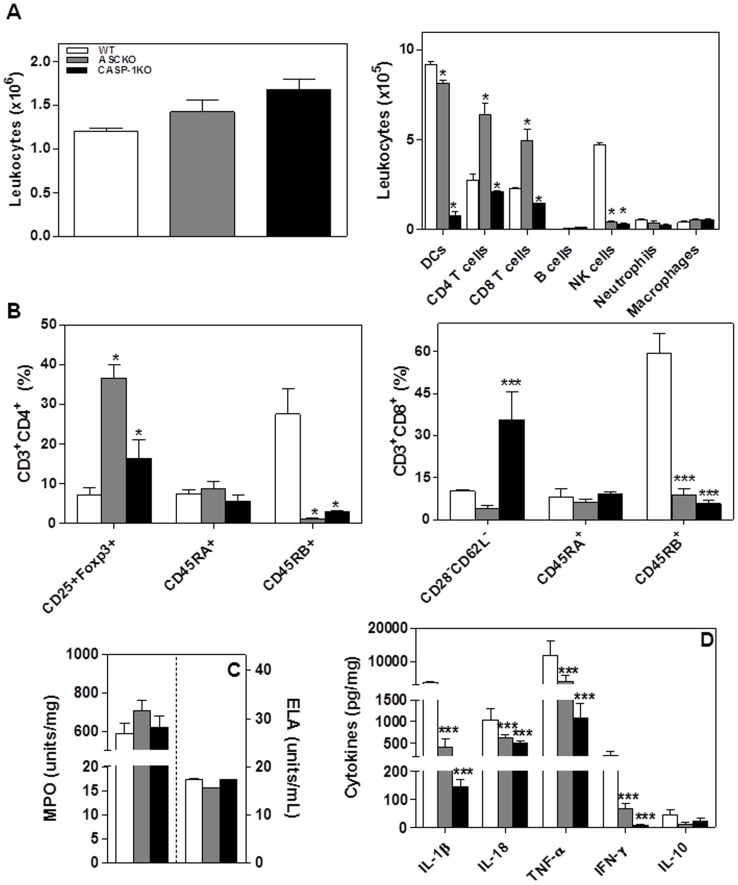
Characterization of SCC lesions in the absence of inflammasome after 20 weeks. (A) The absolute number and phenotype of leukocytes in the tumor lesions were determined by flow cytometry. (B) Flow cytometry analyses of CD25^+^Foxp3^+^, CD45RA^+^, CD45RB^+^ and CD28^−^CD62L^−^ expression on CD4 e CD8 T cells. (C) Myeloperoxidase (MPO) and elastase (ELA) activity in tumor microenvironment. (D) Cytokines profile in tumor microenvironment at 20 weeks after SCC induction. Values are mean ± SEM; *p≤0.05, *** *p*≤0.001 compared with WT mice.

In order to verify whether inflammasome signaling alter immune cells infiltration during SCC development, the population of leukocytes present in lesions was analyzed (Fig. 2). At the 12^th^ week, inflammasome loss of function decreased leukocytes infiltration into the tumor microenvironment [ASC-KO (2.1±0.7×10^6^) and CASP-1-KO (1.4±0.3×10^6^)], as compared to WT mice (2.75±0.16×10^6^) (Fig. 2A). At the 20^th^ week, we found no statistically significant differences in the leukocyte infiltration among the ASC-KO and CASP-1-KOgroups (Fig. 3A). Using FSC and SSC analysis we determined the phenotype of infiltrating cells. Phenotypic analysis of leukocyte population demonstrated significantly decreased T lymphocytes (CD4^+^ and CD8^+^ T cells), DC, NK cells, neutrophils and macrophages infiltration in lesions from ASC-KO and CASP-1-KO mice when compared with the WT group (Fig. 2A). With respect to B cell infiltration, we found no statistically significant differences between the ASC-KO, CASP-1-KO and WT groups (Fig. 2A). At the 20^th^ week, we observed differences in the type of leukocyte infiltrating the lesions (Fig. 3A). Lesions from both ASC-KO and CASP-1-KO exhibited diminished DC infiltration as compared with WT; CASP-1-KO, but not ASC-KO mice, showed significantly decreased CD3^+^CD4^+^, as well as CD3^+^CD8^+^ T cells when compared with WT mice (Fig. 3A). NK1.1^+^ cells were also decreased in the lesions from both ASC-KO and CASP-1-KO groups as compared with WT mice (Fig. 3A). However, we did not observe differences in the B cells, neutrophils and macrophages in the lesions of all groups at the 20^th^ week (Fig. 3A). Altogether, data demonstrate that the loss of inflammasome function influenced on leukocytes infiltration into the tumor microenvironment.

Since CD4^+^CD25^+^ T cells expressing Foxp3 (regulatory T cells) have been related to the progression of SCC [6], [7], the presence of these cells in the tumor was evaluated. At the 12^th^ week, significant increase was found in the CD4^+^CD25^+^Foxp3^+^ T cells infiltrating lesions in the ASC-KO (29.9±13%) and CASP-1-KO (9.7±5%) as compared with WT mice (2.5±1%) (Fig. 2B). Similar results were found at the 20^th^ week (Fig. 3B). Different studies indicate that CD8^+^ T lymphocytes can also exhibit suppressor activity by inhibiting Th1 proliferation [28]–[30]. For this reason, the presence of these cells in the tumor microenvironment was also investigated. The infiltration of suppressor CD8^+^CD28^−^CD62L^−^ T cells (Ts) was significantly increased in ASC-KO as compared to WT mice (Fig. 2B). Different from that seen at the 12^th^ week, CD8^+^CD28^−^CD62L^−^ T cells numbers were increased only in CASP-1-KO lesions at the 20^th^ week (Fig. 3B). Altogether, these data indicate a redirection of immune tumor response in the absence of inflammasome signaling, culminating in tumor suppressor immunity.

In order to determine whether or not the lymphocytes infiltrating SCC lesions presented a memory phenotype, we analyzed percentages of T cells expressing CD45RA and CD45RB [31]–[32]. CD4^+^CD45RB^+^ T cells were detected in lower frequency from tumors samples of mice with inflammasome loss of function at 12^th^ and 20^th^ week (Fig. 2B and 3B). On the other hand, higher frequencies of CD4^+^T cells expressing CD45RA were detected in samples from CASP-1-KO mice at the 12^th^ week as compared with the WT mice (Fig. 2B). In relation to CD8^+^CD45RB^+^T cells, they were detected in lower frequencies in the lesions from ASC-KO mice at the 12^th^ week and 20^th^ week (Fig. 2B and 3B). While we observed an increase of CD8^+^ T cells expressing CD45RB in the lesions from WT at 20^th^ week (Fig. 3B), the percentage of this population still similar KO groups at the 12^th^ and 20^th^ week (Fig. 2B and 3B). No significantly differences were detected in frequencies of CD8^+^CD45RA^+^ T cells (Fig. 2Band 3B). These data show that the absence of inflammasome function clearly decreased the presence of memory T cells infiltrating SCC lesions.

In order to determine whether the tumor-associate neutrophils (TAN) influenced the MPO and ELA activity, which are mediators associated with SCC development [33], we analyzed for the presence of these enzymes in the tumor microenvironment. At 12 weeks, we detected higher activity of MPO in the lesions from WT mice than KO groups and no differences related to ELA were observed among the groups (Fig. 2C). However, we detected similar activity of MPO and ELA in the lesions from all groups at the 20^th^ week (Fig. 3C).

Since the inflammasome cascade is known to control the secretion of IL-1β and IL-18, and both cytokines may influence the activation of immune cells and induce the secretion of Th1 cytokines, the levels of these cytokines were analyzed in the lesions (Fig. 2D and 3D). We found diminished levels of IL-1β, IL-18 and TNF-α levels in lesions from the KO mice when compared with WT mice in both 12 and 20 weeks (Fig. 2D and 3D). The IFN-γ detection was lower only in lesions from CASP-1-KO (Fig. 2D) while levels of IL-10 were similar among all groups at the 12^th^ week (Fig. 2D). At the 20^th^ week, IFN-γ levels were significantly decreased in the lesions from KO mice as compared with WT mice (Fig. 3D). However, levels of IL-10 were similar among all groups at the 20^th^ week (Fig. 3D). These data indicate that the production of cytokines related to an antitumoral response is dependent on inflammasome function.

### Absence of inflammasome modifies the number of cells in the draining lymph nodes

**Figure 4:**
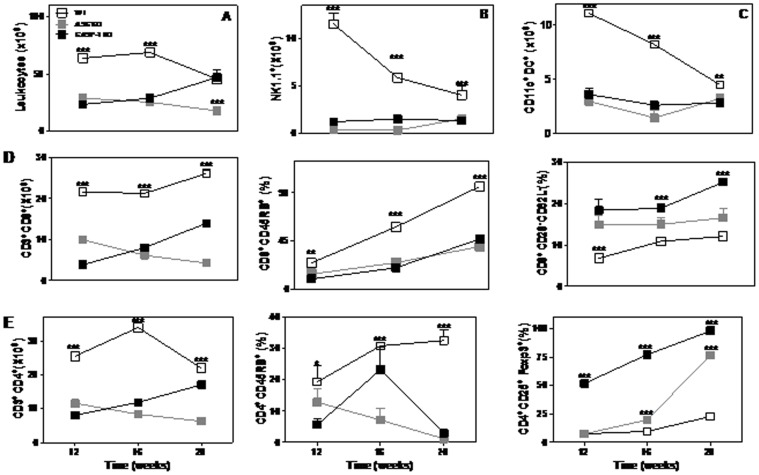
Characterization of leukocyte populations in the lymph nodes with SCC in the absence of inflammasome. The absolute number of leukocytes (A), DC (B), NK (C) CD3^+^CD8^+^ (D), CD3^+^CD4^+^ (E) cells in the lymph nodes was determined by flow cytometry. The proportion of and were determined in samples from ASC-KO (gray square), CASP-1-KO (black square) and WT (white square). Values are mean ± SEM; *** *p*≤0.001 compared with WT mice.

In order to understand if the inflammasome-dependent immune response changes occur only in the tumor microenvironment, the population of leukocytes in the draining lymph nodes (LN) was assessed (Fig. 4). Our data showed that loss of inflammasome function lead to a decreased number of leukocytes in LN during tumorigenesis (Fig. 4A). Lower numbers of NK, DC, CD4 and CD8 T cells were detected in LN from mice with loss of inflammasome function (Fig. 4B–E). CD8 T cells exhibited a suppressor phenotype in the absence of inflammasome function (Fig. 4D). The frequencies of CD4^+^CD45RB^+^ T cells were lower in the LN from KO mice (Fig. 4E). CD4^+^CD25^+^Foxp3^+^ T cells were increasing with tumor development; however, values found in the LN from KO groups were significantly higher as compared with WT mice (Fig. 4E). Altogether, data demonstrate that inflammasome activation can influence in the LN immune response development in the SCC progression.

## Discussion

Data obtained from this study shows the effect of adaptor proteins ASC and the enzyme CASP-1 in the protection against SCC tumorigenesis and progression. Such inflammasome dependence was probably associated with the development of a suppressed immune response impairing the influx of T and NK cells into the lesions as well as an antitumor response in the lymph nodes. In the absence of inflammasome function, SCC lesions' appearance occurred earlier, and the lesions were significantly more abundant. NK cells are potent cytolytic/cytotoxic and cytokine-producing effector cells in response to tumor targets, that is important to modulate the adaptive immune response through cytokines and DC cooperation [14], [34], [35]. NK cells are largely known to have an important role in the tumour immunosurveillance and destruction of aberrant cells [14], [36]. The diminished infiltration of these cells in the SCC lesions as well as in the lymph nodes (data not shown) may be related to the more aggressive disease in the loss of inflammasome function. However, ASC-KO and CASP-1-KO groups had increased NK cells in the spleen at 5^th^ month (data not shown). Therefore, we can speculate that the loss of inflammasome signaling impairs the arrival of NK cells to the tumor lesions, but not their generation. Perhaps, the activation of NK cells in the loss of inflammasome function is impaired along with their reduced infiltration in the lesions since IL-18, as well as IL-1 β, has been shown to activate NK cells [37]. Besides, TNF-α levels were reduced in the absence of inflammasome function. IL-1β and IL-18 are cytokines promoted by inflammasome activation and play an important role in modulating the adaptive immune response, and also mediate T helper cells 1 (Th1)- cytokine secretion [18], [38]. In a separate experiment we noted that NK cells from CASP-1-KO mice were less cytotoxic towards tumor cells (data not shown). This date is in agreement with previous studies showing enhancement of the cytolytic/cytotoxic activity of NK cells is dependent upon cytokines of the IL-1 family [39]. We did not find differences in the activation of NK cells from ASC-KO mice (data not shown) and this result is in agreement with an earlier report [40]. TNF-α and IFN-γ levels also were decreased in lesions from KO mice. In part, this could be explained by the fact that IFN-γ secretion is evoked by IL-18 in antigen- dependent as well as independent pathways [41], [42]. Therefore, decreased levels of IL-18 produced with the absence of inflammasome function might negatively influence IFN-γ secretion. Although TNF-α had been related to chronic inflammation and tumor promotion [43], we have not seen ameliorated symptoms and immune response against cancer from KO mice. Perhaps, TNF-α had a protector role in this SCC model, since this cytokine also have been described as involved with anti-tumor immune response in the presence of IL-1β [43]. Both factors were elevated in the lesions from WT mice compared with KO groups.

Anti-tumor immune responses must begin with the capture of tumour-associated antigens by dendritic cells (DC), either delivered exogenously or captured from dead or dying tumour cells, and migrate to draining lymph nodes [44]. Loss of inflammasome function affected the phenotype of leukocytes infiltrating the tumors (TILs). Herein, we report diminished DC in the lesions from ASC-KO and CASP-1-KO mice. Since the presence of tumor-infiltrating DC has been associated with a favorable prognosis for the patient [44], our data suggest a poor prognosis of the SCC associated with inflammasome loss of function. The cardinal role of DCs in the development of the antitumor immune response is their unique ability to engulf dying tumor cells, a principal source of tumor-associated antigens (TAAs), and cross-present TAAs to CD8^+^ T cells in the LNs [44]. We found diminished DCs as well as CD4^+^ and CD8^+^ T cells in the LNs from mice that had loss of inflammasome function. Therefore, the signaling cascade mediated by the inflammasome activation seems to be associated with DC activation and CD8^+^ T cell differentiation. Our results have shown that CD4^+^ T cells were also decreased in the lesions in the absence of inflammasome function. These Th1 cells are necessary for priming tumor-specific CD8 T cells, influencing the differentiation, expansion of tumor antigen-specific CTLs, and generation/maintenance of long-term CD8^+^ memory T cell responses [45].

Upon antigen encounter, the DCs would also have to receive a suitable activation signal, allowing them to differentiate extensively to promote immunity as opposed to tolerance including enhanced processing and presentation of tumor antigen-derived peptides [46]. Antigen presentation by DCs at the steady state promotes tolerance by Treg production, which would oppose an anti-tumor response [46].Besides the presence of Tregs, increased expression of CD45RA on lymphocytes, resulting in T-cell unresponsiveness, which facilitates immune evasion [6], [7], [12], [46]. Our data also showed increased CD4^+^ T cells expressing CD45RA in the lesions from CASP-1-KO mice compared with WT group. Such differences were not verified in the LN (data not shown), indicating that these cells were increased only in the SCC lesions in the absence of inflammasome components.

Reinforcing the suppression hypothesis, an increase of CD4^+^CD25^+^Foxp3^+^ T cells was found in the SCC lesions and LNs in both KO groups. A previous study related the suppressor T cells and elevated production of IL-10 with the absence of ASC [47]. Although we did not find an increase of IL-10 levels in the absence of inflammasome, the presence of CD4^+^CD25^+^Foxp3^+^ T cells has been currently associated with a poor prognosis in SCC [6], [7]. Although WT mice also exhibited Tregs in the lesions and LN, proportions were decreased when compared with KO groups. Therefore, the absence of inflammasome may possibly be unbalancing the response against SCC to the suppressor immunity.

CD8^+^CD28^−^CD62L^−^ T cells are a group of cells that has been shown to inhibit APC­ mediated T cell activation by direct cell contact­dependent mechanisms [28]–[30]. Although these cells were present in all of the groups, KO mice exhibited increased levels of CD8^+^CD28^−^CD62L^−^ T cells in the lesions and LNs as compared with WT group.

On the other hand, the presence of CD45RB^+^ T cells in lesions and LN was increased with SCC progression in WT but not in KO groups. The infiltration of CD45RO/RB^+^ T cells has been largely associated with an improved prognosis in different types of tumors [48], [49]. Therefore, absence of inflammasome function may also hamper with the development of CD4^+^CD45RB^+^ T cells in the SCC lesions impairing an antitumoral response. Besides their function against several types of tumor, CD45RB^+^ T cells seem to be involved with the inflammasome-IL-1β production [49] that might contribute to the lower levels of this cytokine in the lesions and lymph nodes in the KO mice as compared with WT group.

Although some inflammasomes (NLRC4 and NLRP1) can activate CASP-1 through their CARDs without recruiting ASC; the recruitment of ASC greatly enhances the complex formation and the processing of IL-1β and IL-18 [19], [20], [23]. ASC has also been described acting as a tumor-suppressor in keratinocytes [26]. Besides, it is largely known the role of IL-18 in the activation of Th1 antitumoral immune response [11], [46], [50]. Therefore, other factors found diminished in KO mice can collectively regulate the recruitment of immune cells to the sites of tissue damage and directly act in keratinocytes [51], [52]. Altogether, these events contribute to the susceptibility of these animals to a poor prognostic of the disease in the loss of the inflammasome function. Previous study found opposite results related to CASP-1KO mice [26] but those differences may be resulted from the protocols employed. Differences in the immune response of those mice could not be completely established, allowing the control of the disease in CASP-1KO mice due to low dose of DMBA used to initiate SCC [26]. Although exacerbated inflammation is harmful to immune response against SCC [33] and the absence of IL-1 could control inflammation events [26] without disturbing the development of a Th1 protector immune response. Also, costimulation of TLR2, TLR4 (MyD88), and TLR7/8 enhances IL-1β secretion [53] and all events have been correlated with SCC development [10], [54], [55]. However, we believe that the modulation of the immune response in our investigation was the most responsible for exacerbating the disease, and it is a consequence of an array of alterations from inflammasome loss of function.

In conclusion, our data show that the inflammasome proteins signalling are involved with the protection from chemical-induced squamous cell carcinoma. Inflammatory responses play decisive roles in different stages of tumor development, including initiation, promotion, progression, invasion, and metastasis [33]. We verified that the loss of inflammasome function ablated the development of a protector response against SCC and negatively affected the anti-tumor immune response. Herein we have shown that the inflammasomes perform an important role in the regulation of immune response in carcinogenesis through cell migration of specific immune cell phenotypes, activation of these immune cells and balancing the equilibrium of inflammatory and anti-inflammatory cytokines.
